# Clinical Efficacy and Tolerability of Praziquantel for Intestinal and Urinary Schistosomiasis—A Meta-analysis of Comparative and Non-comparative Clinical Trials

**DOI:** 10.1371/journal.pntd.0003286

**Published:** 2014-11-20

**Authors:** Julien Zwang, Piero L. Olliaro

**Affiliations:** 1 Independent researcher, Bangkok, Thailand; 2 UNICEF/UNDP/WB/WHO Special Programme for Research & Training in Tropical Diseases (TDR), Geneva, Switzerland; 3 Centre for Tropical Medicine, University of Oxford, Oxford, United Kingdom; University of Queensland, Australia

## Abstract

**Background:**

Extensive use of praziquantel for treatment and control of schistosomiasis requires a comprehensive understanding of efficacy and safety of various doses for different *Schistosoma* species.

**Methodology/Principal Findings:**

A systematic review and meta-analysis of comparative and non-comparative trials of praziquantel at any dose for any *Schistosoma* species assessed within two months post-treatment. Of 273 studies identified, 55 were eligible (19,499 subjects treated with praziquantel, control treatment or placebo). Most studied were in school-aged children (64%), *S. mansoni* (58%), and the 40 mg/kg dose (56%); 68% of subjects were in Africa. Efficacy was assessed as cure rate (CR, n = 17,017) and egg reduction rate (ERR, n = 13,007); safety as adverse events (AE) incidence. The WHO-recommended dose of praziquantel 40 mg/kg achieved CRs of 94.7% (95%CI 92.2–98.0) for *S. japonicum*, 77.1% (68.4–85.1) for *S. haematobium*, 76.7% (95%CI 71.9–81.2) for *S. mansoni, and* 63.5% (95%CI 48.2–77.0) for mixed *S. haematobium/S. mansoni* infections. Using a random-effect meta-analysis regression model, a dose-effect for CR was found up to 40 mg/kg for S. *mansoni* and 30 mg/kg for *S. haematobium.* The mean ERR was 95% for *S. japonicum*, 94.1% *for S. haematobium*, and 86.3% for *S. mansoni*. No significant relationship between dose and ERR was detected. Tolerability was assessed in 40 studies (12,435 subjects). On average, 56.9% (95%CI 47.4–67.9) of the subjects receiving praziquantel 40 mg/kg experienced an AE. The incidence of AEs ranged from 2.3% for urticaria to 31.1% for abdominal pain.

**Conclusions/Significance:**

The large number of subjects allows generalizable conclusions despite the inherent limitations of aggregated-data meta-analyses. The choice of praziquantel dose of 40 mg/kg is justified as a reasonable compromise for all species and ages, although in a proportion of sites efficacy may be lower than expected and age effects could not be fully explored.

## Author Summary

Praziquantel is the drug used worldwide to treat intestinal and urinary schistosomiasis, diseases caused by the infection with different species of the parasitic worm *Schistosoma*. Summarizing findings of different studies is important in order to characterize how the parasite responds to treatment and to what extent humans can tolerate the medication. We found over 270 clinical trials on praziquantel, and, although less than one-third could be included in this analysis, the total number of subjects enrolled nears 20,000. This large number of subjects allows deriving general conclusions even though the methodologies used to conduct these studies (how the infection is diagnosed, how treatment effects are assessed) were not always uniform. These analyses confirm that the WHO-recommended praziquantel treatment (single dose of 40 mg/kg) works well on all species and at all ages, although in a proportion of study locations the levels of efficacy may be lower than expected.

## Introduction

Some 779 million people are estimated to live in areas with varying levels of risk of contracting schistosomiasis [1]. The control and treatment of all forms of schistosomiasis is currently based on a single drug, praziquantel (PZQ). The World Health Organization (WHO) recommends that, in areas where the prevalence of infection is sufficiently high not to warrant individual diagnosis, a single dose of 40 mg/kg PZQ be distributed for preventive chemotherapy to either entire communities (through mass treatment) or school-aged children; or, where transmission is low, to be used to treat individuals with demonstrated infection [2]. Of note, while school-aged children are the main target of interventions, also younger children (preschool-aged) are now recognized as a vulnerable population [3], but data for this age group are limited.

PZQ has been available for human use for over three decades, and distributed systematically through preventive chemotherapy from 2006. The cumulative number of treatments has been growing since. Some 34 million have received PZQ in 2010, and seven times more (235 millions) are projected for 2018 [4]; WHO has set target for 75% of the at-risk population to be under regular preventive chemotherapy [5].

With expanding use comes the need to monitor how PZQ performs in different areas, doses, over time and against different *Schistosoma* species. Two Cochrane systematic reviews have analyzed randomized controlled trials of anti-schistosomiasis treatments for *S. haematobium* [6] and *S. mansoni* [7]. A broader, aggregated data meta-analysis including non-comparative studies which did not qualify for the Cochrane reviews was undertaken here to help define more fully the efficacy and safety profile of PZQ across all species causing urinary and intestinal schistosomiasis, including mixed infections. The data generated from this meta-analysis was also intended to be used to help design future clinical investigations, in particular in young children treated with a new paediatric formulation currently developed by a public-private consortium [8].

Efficacy outcomes were measured for the different age-groups and doses and compared between various doses and to other drugs. Similarly, the tolerability profile of PZQ was assessed as incidence of adverse events (AE) and compared between various doses and to other drugs.

## Methods

### Data collection

Published studies were identified by the Cochrane collaboration through electronic searches from January 1, 1990, up to November 2012 of MEDLINE, EMBASE, LILACS, the Cochrane Infectious Diseases Group's trials register and the Cochrane Central Register of Controlled Trials (CENTRAL) using the search term ‘praziquantel’ published in English, French or Portuguese. To qualify for inclusions, patients with a microscopic confirmation of schistosomiasis infection were to be on PZQ mono-therapy at any dosage and dosing regimen, using any formulation and brand; and could be either non-comparative or comparative (randomized controlled trial, quasi-randomized trials). In order to exclude the confounding effect of reinfections, efficacy analysis was restricted to the first 8 weeks post-treatment; hence, otherwise eligible studies with an endpoint beyond 8 weeks were not included in the final analysis.

### Statistical analysis

The aggregated data (as reported in the publications) by species (*S. haematobium, S. mansoni, S. japonicum*, or mixed infections) were extracted from eligible studies of the 273 comparative and non-comparative clinical trials identified through the systematic review. Attrition bias refers to systematic differences between the number of patients at enrolment and at endpoint; it is measured as the number of patients not assessed out of the number of patients enrolled, and is considered high when greater than 10%. Cure rates (CR, defined as the conversion from a positive test pre-treatment to a negative test up to 8 weeks post-treatment) were calculated as provided in the articles. The confidence intervals for the CR were set at 95% (95%CI). The eggs reduction rate (ERR) was defined as the proportional reduction in the mean eggs per gram post-treatment vs. pre-treatment, calculated using geometric or arithmetic means and reported separately depending on how provided in the article. For both outcomes, the endpoint or time of assessment was divided in two groups: within a month (3 to 4 weeks) and between one and two months (5 to 8 weeks).

The Spearman test was used to assess the bivariate correlations between the PZQ dose and CR or ERR in all treatment arms of comparative and non-comparative studies.

Tolerability was assessed by calculating the incidence of adverse events (AE) defined as any sign or symptom occurring after the start of treatment (drug intake), irrespective of whether that sign or symptom was present at baseline or not, of its severity and drug-event relationship. The mean incidence was presented for the PZQ 40 mg/kg treatment groups excluding PZQ 40 mg/kg syrup, and Levo-PZQ 20 mg/kg. Most of the publications did not report the brand name and only two studies compared directly two different brands (Biltricide and Distocide) of PZQ.

The 95%CI for the mean CR, ERR, AE were calculated using a bootstrap resampling method with a maximum of 1000 replicates [9].

For randomized controlled studies assessing the efficacy (CR) and tolerability (AE) of PZQ vs. other drugs, placebo, or comparing different PZQ dosing regimens, risk ratios with 95% confidence intervals (RR, 95%CI), meta-analysis with random effect on the study/site was used and pooled RR presented using the DerSimonian and Laird procedure for random effects models [10]. Heterogeneity was expressed as I^2^ [11].

CR and ERR were log-transformed in multivariate meta-regression to assess the PZQ dose-effect (continuous in mg/kg), along with age (continuous in year), endpoint (continuous in week) and date (continuous in year) with a random intercept for each study/site when the Lagrange multiplier (LM) test was significant to account for heterogeneity.

Graphical displays of comparisons (PZQ vs. comparator groups) and heterogeneity for CR and ERR were illustrated using Forest plots [12].

Age groups were categorized as (i) preschool-aged children (<6 years old), (ii) school-aged children (6–19 years old), (iii) adults (20 years old or more), or (iv) all ages if age-specific data could not be extracted. The sample size (number of subjects by site), the endpoint (weeks), the intensity of infection at baseline (egg counts before treatment) were presented according to the *Schistosoma* species.

Data were analyzed using Stata v11 (Stata Corp.). The PRISMA (Preferred Reporting Items for Systematic reviews and Meta-Analyses) statement [13] was used as a guide in the reporting of this study.

## Results

### Study characteristics

Of the 273 published studies identified by systematic search of the literature, 92 were PZQ treatment trials; of these, 37 studies had an endpoint for efficacy beyond 2 months and were excluded, leaving 55 studies (41 comparative, 14 non-comparative) with an endpoint within 8 weeks [14]–[68].

The first eligible study was published in 1979, and half of the studies were conducted by 1998. The studies enrolled a total of 19,499 subjects in 189 treatment arms. The median study size was 206 patients (range 43–1,540). Attrition was acceptable (9%, n = 17,718), leaving 91% of the subjects with efficacy outcomes at the time of the study endpoint; of these, 42% were assessed within 4 weeks in 30 studies, and 58% between 5–8 weeks in 25 studies. More subjects were assessed between 5–8 weeks for *S. mansoni* (65%) and *S. japonicum* (71%), while more were assessed on week 3–4 for *S. haematobium* in 56% of cases and mixed *S. mansoni/haematobium* infections in 71% of cases.

PZQ contributed to 74% (n = 13,048) and comparator drug or placebo to 26% (n = 4,670) of all subjects with outcomes. Of the subjects treated with PZQ at doses comprised between 10 and 60 mg/kg, PZQ 40 mg/kg was the most frequent dose (56%, n = 9,990).

**Figure 1:**
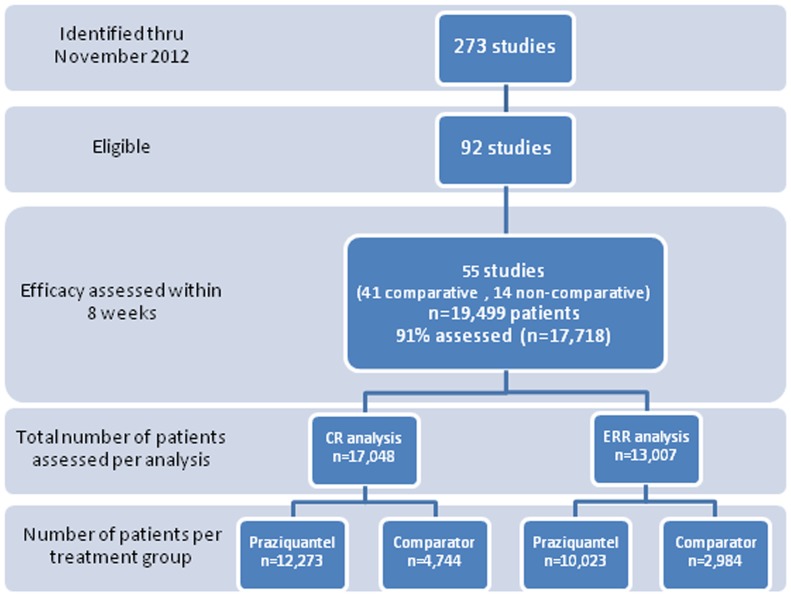
Flow chart of the number of studies and patients screened and eligible for the efficacy analyses of cure rate (CR) and egg reduction rates (ERR). Of the 41 comparative studies identified, 19 directly compared different doses and schedules of PZQ; 7 compared praziquantel with artesunate combined with praziquantel, sulfalene, sulfamethoxypyrazine/pyrimethamine, or mefloquine; 6 with artesunate alone; 3 with metrifonate; 1 with nitrifonate and 1 with metrifonate+nitrifonate; 6 with oxamniquine, 4 with oltipraz, 1 with albendazole, 1 with mefloquine, and 3 with PZQ in combination with artemether, albendazole, or metrifonate.

CR was assessed in 17,017 subjects (of whom 12,273 (69%) treated with PZQ) and ERR in 13,007 subjects (77%, n = 10,023 on PZQ) (Figure 1).

Studies were conducted in 24 countries and 82 sites. The largest population was from the WHO AFRO region (13,251 subjects, 68%), followed by EMRO (Egypt, Sudan and Saudi Arabia, 23%). The largest groups were *S. mansoni* subjects enrolled in Egypt (n = 2,606, 13.4%), Kenya (11.9%), Sudan (9.0%) and Uganda (6.6%) (Table S1).

The risk of attrition bias was low in 57%, high in 38% and not assessable in 5%. The risk was high in 45% (10/22) of the community-based studies and 31% (10/32) of the school-based studies. The assessment of bias and the main characteristics of these studies are summarized in Table 1 and Table S2.

**Table 1:** Main characteristics of the studies included – *S. haematobium* (sh), *S. japonicum* (sj) and mixed infections *S. haematobium-S. intercalatum* (si).

Publication	Country	End point	Species	Age range	Total (n)	PZQ referent		Comparator		Screening method
						dose	n	drug	n	sample(n)*day(n)
Bornmann 2001	Gabon	2	sh	5–13	296	pzq40	89	other	207	1*1
Burchard 1984	Gabon	2	sh	5–14	138	pzq60	65	other	73	1*1 dup
Davis 1981	Zambia	1	sh	7–17	151	pzq40	45	pzq20-60	106	1*3
de Clercq 2002	Senegal	2	sh	7–14	267	pzq40	133	other	134	1*2
Inyang-Etoh 2008	Nigeria	2	sh	4–20	174	pzq40	42	pzq40 + other	220	2*2
Keiser 2010	Ivory coast	1	sh	8–16	83	pzq40	26	other	57	2*1
King 2002	Kenya	2	sh	4–23	200	pzq40	101	pzq20	99	1*2
Latham 1990	Kenya	2	sh	7–15	48	pzq40	16	other	32	1*1
McMahon 1983	Tanzania	2	sh	1–60*	77	pzq30	30	other	47	1*2
McMahon 1979	Tanzania	1	sh	7–15	125	pzq40	65	pzq30 and placebo	60	3*3
Midzi 2008	Zimbabwe	2	sh	2–19	624	pzq40	624			1*3
N'goran 2003	Ivory coast	1	sh	5–15	354	pzq40	354			1*1 dup
Olds 1999	Kenya	2	sh	6–19	380	pzq40	95	other	285	2*1
Oyideran 1981	Nigeria	1	sh	7–13	82	pzq40	40	pzq30 and placebo	42	1*3
Rey 1983	Niger	1	sh	15–19	188	pzq40	54	pzq30 and other	134	2*2
Sissoko 2009	Mali	1	sh	6–15	781	pzq40	389	other	392	1*1 dup
Tchuente 2004	Cameroon	1	sh	school	515	pzq40	515			1*2
Wilkins 1987	Gambia	1	sh	5–17	619	pzq40	143	pzq10-20 and other	476	1*1
Kern 1984	Gabon	2	sh + si	10–17	158	pzq60	77	other	81	1*2
Belizario 2007	Philippines	1	sj	10–19	203	pzq40	102	pzq60 and other	101	2*2
Hou 2008	China	2	sj	10–60	196	pzq60	55	other	141	2*1 tri
Olds 1999	Phillipines, China	2	sj	5–16	793	pzq40	203	other and placebo	590	2*1
Olliaro 2011	Philippines	1	sj	7–12	200	pzq40	101	pzq60	99	1*2 dup
Abu elyazed 1998	Egypt	2	sm	5–50	939	pzq40	551	pzq60	388	3*3
Barakat 2005	Egypt	1	sm	5–39	83	pzq40	38	other	45	1*2
Berhe 1999	Ethiopia	2	sm	5–17	541	pzq40	541			1*1
Botros 2005	Egypt	2	sm	7–73	271	Pzq40	165	other	106	1*3
daSilva 1986	Brazil	1	sm	14–60*	94	pzq55	48	other	46	3*1
Declerq 2000	Senegal	2	sm	6–61	156	pzq40	39	other	117	1*1 dup
Declerq tmih 2000	Senegal	2	sm	1–50	110	pzq40	36	other	74	1*1
Degu 2002	Ethiopia	2	sm	10–14	148	pzq40	148			1*1
Friis 1988	Botswana	2	sm	school	81	pzq40	81			1*1 dup
Ghandour 1995	Saudi Arabia	1	sm	1–50	170	pzq40	170			na
Gryseels 1987	Burundi	2	sm	<20 and ≥20	1138	pzq40	272	pzq20-30 other	866	1*1 dup
Guisse 1987	Senegal	1	sm	5–15	130	pzq40	67	pzq60	63	2*2
Homeida 1989	Sudan	2	sm	1–60*	806	pzq40	400	pzq40 brand2	406	1*1
Ismail 1994	Egypt	2	sm	6–18	463	pzq40	463			1*1
Kabatereine 2003	Uganda	2	sm	5–50*	482	pzq40	482			3*1
Kardaman 1983	Sudan	1	sm	5–60*	388	pzq40	388			2*1
Massoud 1984	Egypt	1	sm	school	179	pzq40	59	pzq10-20	120	1*1
McMahon 1981	Tanzania	1	sm	1–60*	91	pzq40	49	pzq50	42	1*3
Metwally 1995	Egypt	1	sm	8–16	366	pzq40	149	pzq20	217	3*3 tri
Mohamed 2009	Sudan	1	sm	8–17	92	pzq40	46	other	46	1*2
Navaratnam 2012	Uganda	1	sm	1–5	297	pzq40	149	syrup pzq40	148	3*1
Obonyo 2010	Kenya	1	sm	7–12	212	pzq40	106	other	106	1*1 dup
Olds 1999	Kenya	2	sm	6–19	367	pzq40	82	other placebo	285	2*1
Olliaro 2011	Brazil	1	sm	10–19	190	pzq40	96	pzq60	94	1*2 dup
Olliaro 2011	Mauritania	1	sm	10–19	185	pzq40	92	pzq60	93	1*2 dup
Olliaro 2011	Tanzania	1	sm	10–19	244	pzq40	119	pzq60	125	1*2 dup
Raso 2004	Ivory coast	2	sm	1–60*	161	pzq40	161			3*1
Simonsen 1990	Ethiopia	1	sm	5–14	206	pzq40	206			2*1
Sousa-Figueiredo 2012	Uganda	1	sm	1–7	369	pzq40	369			1*2
Stelma 1997	Senegal	2	sm	5–75	86	pzq40	44	other	42	2*2
Taddese 1988	Ethiopia	1	sm	17–52	194	pzq40	99	other	95	1*1
Teesdale 1984	Malawi	1	sm	9–15	69	pzq40	18	other	51	4*1
Thiongo'o 2002	Kenya	2	sm	5–17	1018	pzq40	526	pzq60 and other	492	1*3 dup
Utzinger 2000	Ivory coast	1	sm	6–14	194	pzq60	194			1*4
El Tayeb 1988	Sudan	1	sm+sh	7–12	111	pzq40	54	other	57	1*2
Kardaman 1983	Sudan	1	sm+sh	5–60*	43	pzq40	43			2*1
Kardaman 1985	Sudan	2	sm+sh	7–11	211	pzq40	211			1*1
Taylor 1988	Zimbabwe	1	sm+sh	10–15	373	pzq40	77	pzq10-20-30 and placebo	296	3*1

The largest number of subjects with efficacy outcomes was for a *S*. *mansoni* infection (57.8%), followed by *S. haematobium* (29.3%), *S. japonicum* (7.9%) and mixed infections (5%). Most of the subjects were school-aged children (63.8%); preschool-aged children accounted for 2.9%, adults 4.7%, and subjects of all ages 28.6% (Table S3). Two studies including all age's subjects also specified age categories, including schoolchildren [36], [66].

### Laboratory diagnosis

To diagnose and quantify the infection, the trials on *S. haematobium* used the filtration method with up to two specimens in duplicates over three days except in one study using reagent strips, while trials on *S. mansoni* used the Kato-Katz technique with up to three specimens over three days in triplicates (Table S4).

Egg counts were reported using different approaches (number of specimens and tests) for the different intestinal or urinary schistosomiasis species for 13,135 subjects. The mean egg count before treatment was 910 (95%CI 369–1642) and 251 (95%CI 201–307) eggs per gram of feces for *S. mansoni*, in studies using arithmetic or geometric means, respectively; 178 (95%CI 95–274) eggs per gram of feces for *S. japonicum*; and 125 (95%CI 60–196) and 137 (95%CI 70–226) eggs per mL of urine for *S*. *haematobium*, for arithmetic and geometric means, respectively.

### Efficacy

In subjects treated with PZQ, the efficacy of PZQ in any species (n = 13,105) was measured in 508 (4%) preschool, 7,776 (59%) school-aged children, 428 (3%) adults, and 4,393 (34%) subjects of all ages.

The number of treatment arms with different doses of PZQ varied greatly; the 40 mg/kg dose was by far the most common (66%, 77/117), followed by the 60 mg/kg dose (14%, 16/117). All doses were not tested on each and every species or age groups. The only dose administered in preschool-aged children was 40 mg/kg for *S. mansoni*; school-aged children received doses ranging 10–60 mg/kg (72% were on 40 mg/kg); adults received 20–40 mg/kg; studies on all-age subjects administered doses ranging 20–60 mg/kg (76% were on 30 mg/kg).

#### Cure rates (CR) with PZQ

**Figure 2:**
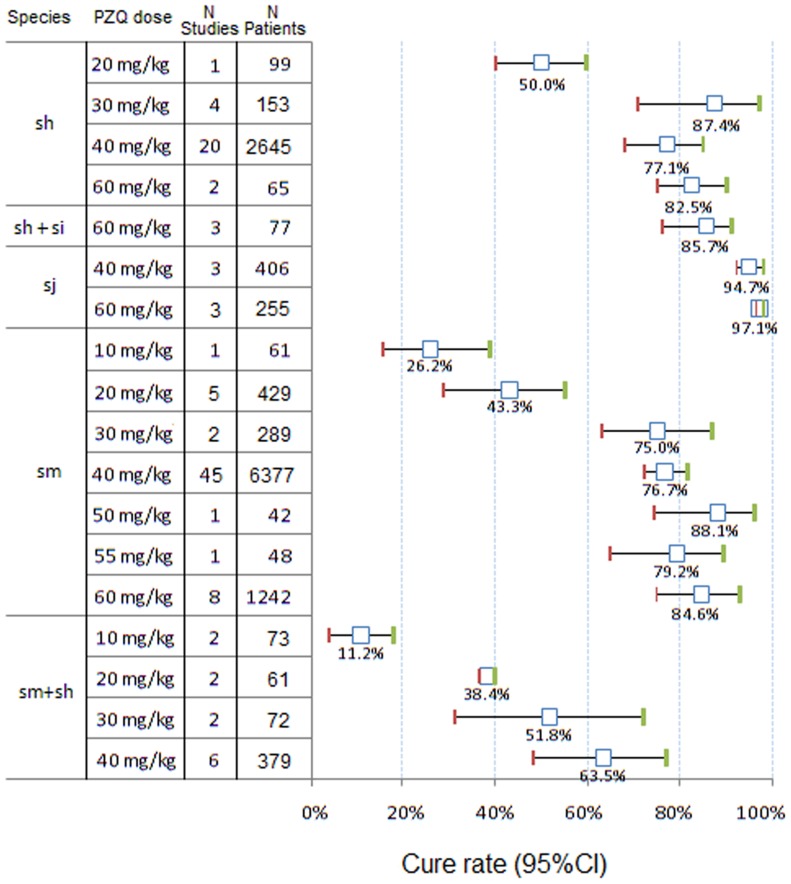
Forest plot of praziquantel (PZQ) cure rates with 95% CIs by species and dose (all age groups). sh, *S. haematobium*; si, *S. intercalatum*; sj, *S. japonicum*; sm, *S. mansoni*.

Mean dose-specific CRs with 95%CIs by species are presented in Figure 2. CRs for any dose of PZQ appeared to be highest in *S. japonicum* infections (40 and 60 mg/kg); and were higher in *S. haematobium*, mixed *S. haematobium/intercalatum* and *S. mansoni* infections than in pure and mixed *S. mansoni/haematobium* infections.

The recommended dose of 40 mg/kg achieved CRs of 94.7% (95% CI 92.2–98.0) for *S. japonicum*, while it was 77.1% (95% CI 68.4–85.1) for *S. haematobium*, 76.7% (95% CI 71.9–81.2) for *S. mansoni, and* 63.5% (95% CI 48.2–77.0) for mixed *S. haematobium* and *S. mansoni* infections.

#### Dose-effect analysis

There was a significant relationship (Spearman test) between the CRs in subjects treated for *S. mansoni* and the PZQ dose: from 26.2% with PZQ 10 mg/kg to 84.6% with PZQ 60 mg/kg (r = 0.434, p = 0.001), as well as for mixed *S. mansoni + S. haematobium* infections (r = 0.764, p = 0.001) but not for *S. haematobium* (r = 0.019, p = 0.923) nor for *S. japonicum* (r = 0.396, p = 0.437).

#### Endpoint analysis

PZQ 40 mg/kg CRs assessed on week 3–4 were 82.7% (95%CI 70.3–92.9) and on week 5–8 were 69.9% (95%CI 58–78.7) for S. *haematobium*, and were 79.6% (95%CI 72.8–85.7) and 73.9% (95%CI 67.1–80.6) for S. *mansoni*, respectively. Although a direct comparison is not possible, 95%CIs overlap for both species.

#### CR with PZQ 40 mg/kg vs. comparators

**Figure 3:**
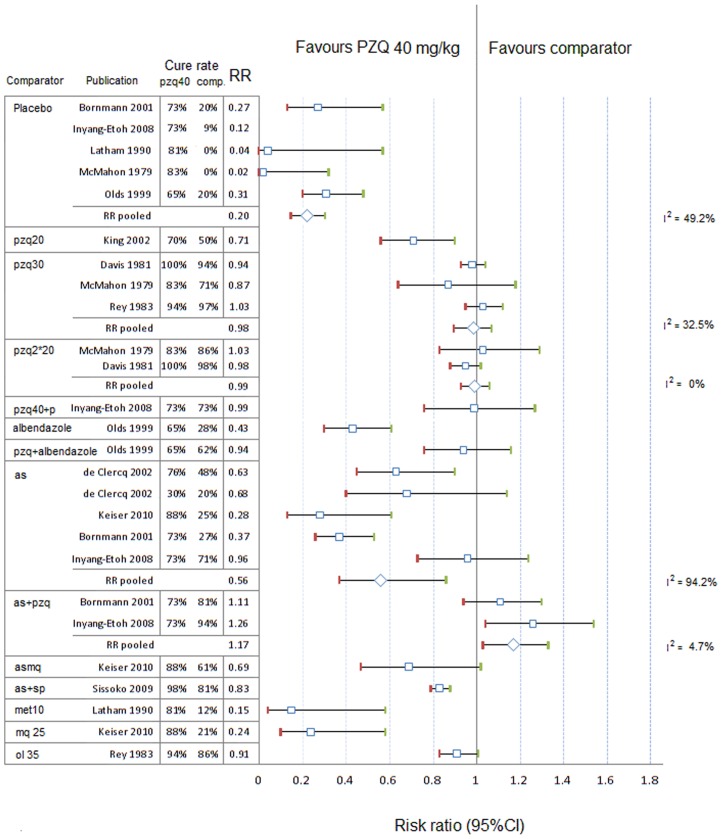
Forest plot of relative risks of cure rates with 95%CIs, *S. haematobium*, PZQ 40 mg/kg vs. comparators. as, artesunate; ol, oltipraz; pzq, praziquantel; sp, sulfadoxine-pyrimethamine; met, metrifonate, mq, mefloquine; p, placebo; comp, comparator; unit next to the drug: dose in mg/kg; RR, risk ratio; I^2^ (Higgins' I squared) is calculated for pooled subgroups as  =  100%*(*Q* - df)/*Q*, where *Q* is Cochran's heterogeneity statistic and df the degrees of freedom.

**Figure 4:**
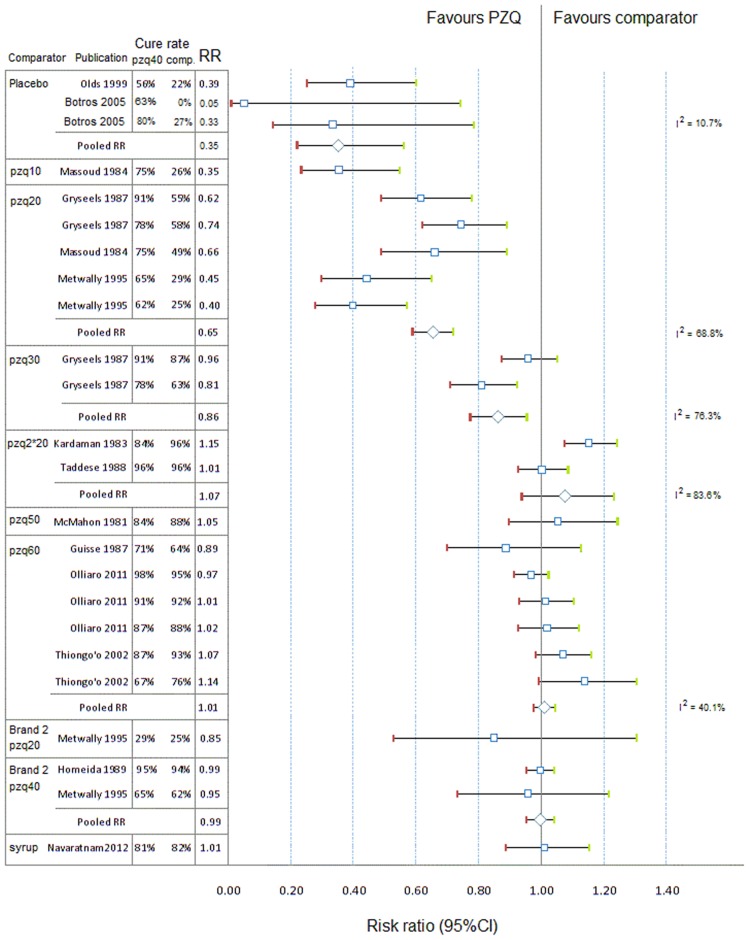
Forest plot of risk ratios of cure rates with 95%CIs, *S. mansoni*, PZQ 40 mg/kg vs. other PZQ regimens. comp, comparator; ci, confidence interval; pzq, praziquantel; unit next to the drug: dose in mg/kg; RR, risk ratio; I^2^ (Higgins' I squared) is calculated for pooled subgroups as  =  100%×(*Q* - df)/*Q*, where *Q* is Cochran's heterogeneity statistic and df the degrees of freedom.

**Figure 5:**
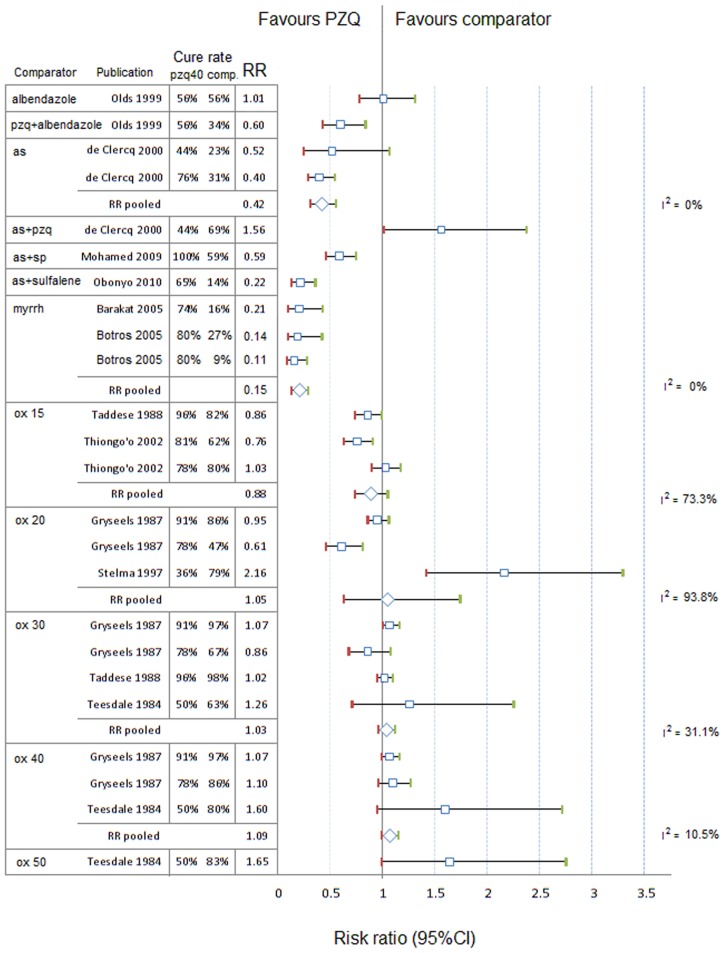
Forest plot of risk ratio of cure rates with 95%CIs, S. mansoni, PZQ 40 mg/kg vs. other regimens. as, artesunate; ox, oxamniquine; pzq, praziquantel; sp, sulfadoxine-pyrimethamine; mq, mefloquine; comp, comparator; ci, confidence interval; unit next to the drug: dose in mg/kg; RR, risk ratio; I^2^ (Higgins' I squared) is calculated for pooled subgroups as  =  100%×(*Q* - df)/*Q*, where *Q* is Cochran's heterogeneity statistic and df the degrees of freedom.

The RR (95%CI) of CR between praziquantel 40 mg/kg and other PZQ regimens, placebo or other treatments are presented in Figure 3 for *S. haematobium* and Figures 4 and 5 for *S. mansoni*.

Using meta-analysis regression model with random effect on the sites, the CR for treating *S. haematobium* with praziquantel 40 mg/kg was higher than praziquantel 20 mg/kg (RR = 0.71, 95%CI 0.56–0.90, p = 0.004) and not different from praziquantel 30 mg/kg (p = 0.575); PZQ 40 mg/kg had higher CR than artesunate alone (RR = 0.55, 95%CI 0.36–0.83, p = 0.005) or in combinations, mefloquine alone, and metrifonate 10 mg/kg (RR = 0.15, 95%CI 0.04–0.58, p = 0.001).

On S. *mansoni*, using similar methods, the CR of PZQ 40 mg/kg was higher than PZQ 20 mg/kg (RR = 0.65, 95%CI 0.59–0.72, p = 0.001), PZQ 30 mg/kg (RR = 0.89, 95%CI 0.75–0.95, p = 0.004), and not different from higher doses (50 mg/kg, p = 0.544; 60 mg/kg, p = 0.477); the CR for PZQ 40 mg/kg was significantly higher than artesunate and combinations, and myrrh (p = 0.001 for all comparisons); not different from oxamniquine 15, 20, 30 mg/kg; slightly lower than oxamniquine 40 mg/kg (RR = 1.09, 95%CI 1.01–0.18, p = 0.034), but not significantly different from oxamniquine 50 mg/kg (RR = 1.65, 95%CI 0.99–2.75, p = 0.056).

On S. *japonicum*, using similar methods, the CR of PZQ 40 mg/kg was not different from PZQ 60 mg/kg (RR 1.02, 95%CI 0.97–1.07, p = 0.461), and higher than placebo (p = 0.001).

On mixed S. *haematobium* and *mansoni*, the CR of PZQ 40 mg/kg was not significantly higher from lower PZQ dose (10 mg/kg: RR 0.15, p = 0.060; 20 mg/kg RR 0.63, p = 0.135; 30 mg/kg RR 0.86, p = 0.278).

#### Eggs reduction rate (ERR)

**Figure 6:**
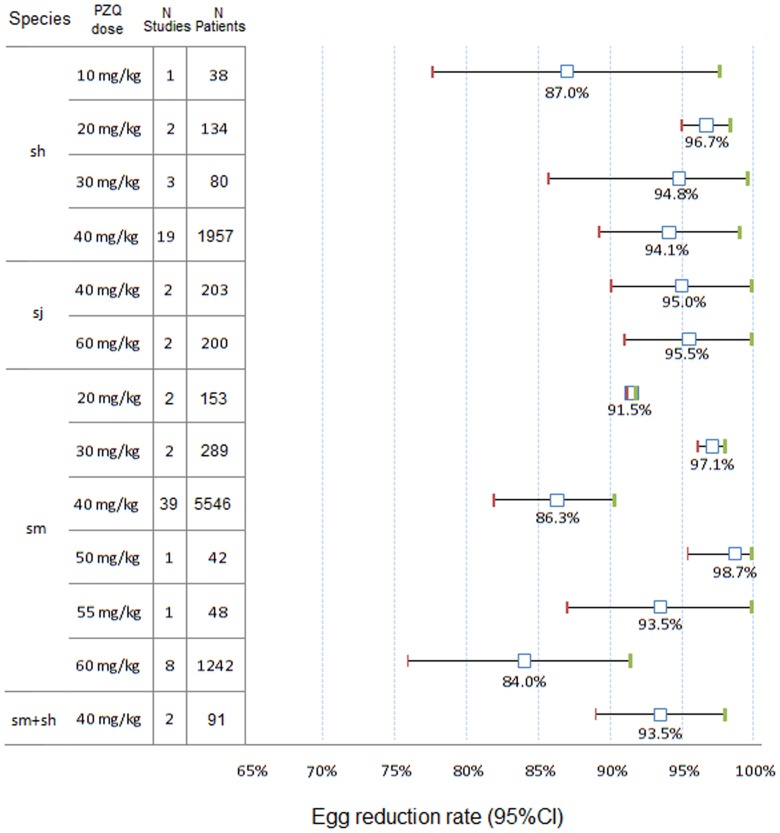
PZQ egg reduction rates with 95% CIs by species and dose. ci, confidence interval; sh, *S. haematobium*; si, *S. intercalatum*; sj, *S. japonicum*; sm, *S. mansoni*.

The ERR was measured for 13,007 subjects in 126 study/sites. ERR by species and PZQ dose from non-comparative and comparative trials are presented in Figure 6.

The mean ERR was over 90% in subjects of any age treated with PZQ doses greater than 10 mg/kg for *S. haematobium* and 87% or more for *S. mansoni* and 89% or more for *S. mansoni/haematobium* mixed infections (40 mg/kg); for *S. japonicum*, the ERR was ∼95% (40 and 60 mg/kg).

There was no significant relationship (Spearman test) between the ERRs in subjects treated with any PZQ dose and species: *S. mansoni* (r = −0.126, p = 0.370), *S. haematobium* (r = 0.057, p = 0.786), as well as for S. *japonicum* (r = 0.236, p = 0.764).

With PZQ 40 mg/kg, the ERR assessed was 94.6% (95%CI 89.9–98.0) on week 3–4 and 93.4% (95%CI 83.2–100) on week 5–8 for S. *haematobium*, for S. *mansoni*, the ERR was 87.4% (95%CI 82.7–91.5) and 72.0% (89.0%, 95%CI 83.7–94.2) respectively.

More details on efficacy rates by age groups and dose are given in Table 2.

**Table 2:** Cure rates (CR) and egg reduction rates (ERR) with 95% confidence intervals (95%CI) calculated by boot-strapping by age-group, species and praziquantel dose.

Age group	Species	PZQ dose (mg/kg)	Cure rate (CR)							Egg reduction rate (ERR)						
			CR	Lower 95%CI	Upper 95%CI	N patients	N treatment arms	% assessed within 1 month	Endpoint (median week)	ERR	Lower 95%CI	Upper 95%CI	N patients	N treatment arms	% assessed within 1 month	Endpoint (median week)
preschool	sm	40	69.0%	56.4%	81.7%	414	2	100%	4	85.6%	82.2%	89.0%	414	2	100%	4
school-aged	sh	10								87.0%	77.6%	97.6%	38	1	100%	4
		20	98.1%	98.1%	98.1%	53	1	100%	4	98.4%	94.4%	99.9%	35	1	100%	4
		30	71.0%	71.0%	71.0%	31	1	100%	4	92.6%	85.7%	99.6%	50	2	100%	4
		40	76.6%	67.8%	85.2%	2490	18	56%	4	93.9%	89.1%	98.7%	1856	18	67%	4
		60	82.5%	75.0%	90.0%	65	2	0%	6							
	sh + si	60	85.6%	76.0%	91.0%	77	3	0%	5							
	sj	40	94.7%	92.2%	98.0%	406	3	67%	3	95.0%	90.1%	99.9%	203	2	100%	3
		60	97.5%	97.0%	98.0%	200	2	100%	3	95.4%	91.0%	99.9%	200	2	100%	3
	sm	10	26.2%	26.2%	26.2%	61	1	100%	4							
		20	40.3%	24.9%	58.0%	376	4	75%	4	91.7%	86.7%	97.3%	100	1	0%	6
		30	63.0%	63.0%	63.0%	187	1	0%	6	96.1%	93.5%	98.8%	187	1	0%	6
		40	74.6%	68.3%	80.6%	2340	19	47%	5	89.1%	83.3%	94.2%	1856	18	43%	5
		60	78.6%	67.8%	90.6%	667	5	60%	4	84.2%	73.5%	94.2%	667	5	60%	4
	sm+sh	10	11.1%	4.2%	18.1%	73	2	100%	4							
		20	38.3%	36.7%	40.0%	61	2	100%	4							
		30	51.8%	31.4%	72.2%	72	2	100%	4							
		40	67.6%	52.4%	81.2%	342	5	60%	4	98.0%	87.1%	99.7%	54	1	100%	4
adult	sh	30	97.4%	97.4%	97.4%	39	1	100%	4							
		40	94.4%	94.4%	94.4%	54	1	100%	4							
	sm	20	55.0%	55.0%	55.0%	53	1	0%	6	91.2%	84.7%	98.2%	53	1	0%	6
		30	87.0%	87.0%	87.0%	102	1	0%	6	98.0%	95.5%	99.9%	102	1	0%	6
		40	94.2%	91.0%	96.0%	180	3	67%	4	77.0%	63.0%	98.2%	180	3	67%	4
all ages	sh	20	50.0%	50.0%	50.0%	99	1	0%	6	95.0%	90.9%	99.3%	99	1	0%	6
		30	87.0%	87.0%	87.0%	30	1	0%	8	99.0%	95.5%	99.9%	30	1	0%	8
		40	70.0%	70.0%	70.0%	101	1	0%	6	98.0%	95.3%	99.9%	101	1	0%	6
	sj	60	96.4%	96.4%	96.4%	55	1	0%	6							
	sm	40	76.7%	67.7%	84.5%	3443	20	40%	5	85.5%	79.0%	91.4%	3443	20	40%	5
		50	88.1%	88.1%	88.1%	42	1	100%	4	98.7%	95.4%	99.9%	42	1	100%	4
		55	79.2%	79.2%	79.2%	48	1	100%	4	93.5%	87.0%	99.9%	48	1	100%	4
		60	94.4%	92.5%	95.9%	575	3	67%	3	83.5%	68.7%	92.0%	575	3	67%	3
	sm+sh	40	43.2%	43.2%	43.2%	37	1	100%	4	89.0%	80.6%	98.3%	37	1	100%	4

Legend: sh, *S. haematobium*; si, *S. intercalatum*; sj, *S. japonicum*; sm, *S. mansoni*.

#### Brand analysis

Both brands (Biltricide and Distocide) of PZQ 40 mg/kg were effective in reducing infection intensity (ERR was 99.5% for both groups)[31]; similarly, there was no difference in CR with either 40 mg/kg (pooled RR 0.99, 95%CI 0.96–1.03, p = 0.745)[31], [46], or 20 mg/kg (RR 0.85, 95%CI 0.54–1.31, p = 0.453)[31].

### Tolerability

#### Adverse events (AEs)

Of the 273 published studies identified, signs and symptoms recorded within 48 hours of treatment were reported in 12,435 subjects enrolled in 40 studies: 25 studies from the efficacy analysis, contributing to 75% of the subjects assessed for tolerability (n = 9,151) and 15 additional studies, (n = 3,284) [69]–[83], meaning that 45% of the studies eligible for the efficacy meta-analysis reported on tolerability. Ninety-six (96) treatment arms were analyzed of which 64 were PZQ administered from 20 to 80 mg/kg. Most of the recorded AEs were gastro-intestinal, neurological and dermatological (Figure S1).

On average the incidence of subjects experiencing at least one AE was 56.9% (95%CI 47.4–67.9) in twelve studies reporting this tolerability outcome and treating 2,027 subjects with PZQ 40 mg/kg (all brands). The incidence of specific AEs ranged from 2.3% for urticaria to 31.1% for abdominal pain (Table 3) – detailed below.

**Table 3:** Adverse event incidence, praziquantel 40 mg/kg.

Adverse event	Number		Incidence	95%CI Bootstrap	
	Studies	Patients	(%)	Lower bound	Upper bound
Any adverse event	13	2272	56.0	45.2	66.4
Abdominal pain	30	6212	31.1	22.0	39.0
Muscle pain	2	129	29.2	10.0	48.0
Joint pain	3	642	25.7	7.4	59.0
Dizziness	30	6328	13.9	9.1	19.0
Headache	27	5642	13.7	9.1	18.0
Diarrhea	27	5790	12.7	8.0	17.0
Fatigue	10	2279	11.6	5.4	18.0
nausea	22	5508	10.6	6.9	14.0
Itching/rash	15	2885	10.4	3.9	19.0
Weakness	5	882	10.0	3.7	17.0
Haematuria	3	727	9.6	0.0	23.0
Vertigo	3	304	8.7	3.8	14.0
Vomiting	26	5339	7.2	4.8	9.7

Legend. ci, confidence interval.

#### AEs with PZQ 40 mg/k vs. comparators

In comparative studies, and using meta-regression with random effect on the study/site, subjects treated with PZQ 40 mg/kg were at lower risk for any AE compared to PZQ 60 mg/kg (RR 0.73, 95%CI 0.59–0.90, p = 0.003), oxamniquine 25 mg/kg (RR 0.63, 95%CI 0.50–0.78, p = 0.001), metrifonate 3*10 mg/kg (RR 0.73, 95%CI 0.55–0.98, p = 0.036), while they were at higher risk compared to L-PZQ (RR 1.31, 95%CI 1.05–1.63, p = 0.018) and AS+SP (RR 2.26, 95%CI 1.50–3.41, p = 0.004); there was no difference between 40 mg/kg and PZQ doses (20 mg/kg, 30 mg/kg, 2*20 mg/kg), metrifonate 10 mg/kg, metrifonate 10 mg/kg + niridazole 25 mg/kg. When different brands were compared, Biltricide had more AEs than Distocide (RR 1.50, 95%CI 1.31–1.72, p = 0.001).

The most frequent AEs are listed below by decreasing frequency in PZQ 40 mg/kg recipients.

The incidence of abdominal pain was 31.8% (95%CI 24.4–39.9) in 6,495 subjects treated with PZQ 40 mg/kg in 30 treatment arms. Subject treated with PZQ 40 mg/kg were at higher risks for abdominal pain than PZQ 20 mg/kg (RR = 1.80, 95%CI 1.31–2.48, p = 0.001), metrifonate 10 mg/kg (RR = 1.50, 95%CI 1.21–1.86, p = 0.001), AS+SP (RR = 3.32, 95%CI 1.70–6.49, p = 0.001); while there was no significant difference between PZQ 40 mg/kg and PZQ at various dose (60 mg/kg, 30 mg/kg, 2*20 mg/kg, 2*25 mg/kg, 2*15 mg/kg, 2*35 mg/kg, 2*30 mg/kg, syrup 40 mg/kg), L-PZQ, mefloquine, AS, ASMQ, ASSP, metrifonate 30 mg/kg, nirifonate 150 mg/kg, metrifonate 10mg/kg + nirifonate 250 mg/kg. Divergent results were found when PZQ was compared to oxamniquine: in a study, subjects treated with PZQ 40 mg/kg were at lower risks (RR = 0.48, 95%CI 0.28–0.83, p = 0.001) than oxamniquine 25 mg/kg, while in another study they were at higher risks compared to oxamniquine at 15, 20, 30, 40 mg/kg (p<0.05). Subjects treated with Biltricide were at higher risk of abdominal pain than those treated with Distocide (RR = 2.34, 95%CI 1.74–3.14, p = 0.001).

Muscle pain was reported in 29.2% (95%CI 10.0–48.0) of the 129 subjects receiving PZQ 40 mg/kg at two study/sites and not different from PZQ 2*30 mg/kg. No difference was detected either in two other studies comparing PZQ 55 mg/kg and oxamniquine 15 mg/kg.

Joint pain was reported in 20.2% (95%CI 4.9–42.3) of the 1,097 subjects enrolled in four PZQ 40 mg/kg treatment arms. In comparative studies no difference was detected with metrifonate 10 mg/kg and oxamniquine 25 mg/kg; subjects treated with PZQ 40 mg/kg Distocide (3.7%) were at lower risk compared to PZQ 40 mg/kg Biltricide brand (7.4%, RR 0.50, 95%CI 0.28–0.89, p = 0.018).

Headache was reported in 13.6% (95%CI 9.3–18.6) of the 5,958 PZQ 40 mg/kg recipients enrolled in 27 treatment arms. Subjects treated with PZQ 40 mg/kg were at lower risks than those on oxamniquine 20 mg/kg (RR 0.31, 95%CI 0.11–0.89, p = 0.020), oxamniquine 2*15 mg/kg (RR 9.00, 95%CI 1.18–68.42, p = 0.034) while no difference was detected in other studies vs. other dose of PZQ (from 20 up to 60 mg/kg, syrup 40 mg/kg or L-PZQ), artesunate and combinations, mefloquine, niridazole, or metrifonate.

The incidence of diarrhea was 12.9% (95%CI 8.6–17.9) in 6,106 PZQ 40 mg/kg recipients enrolled in 27 treatment arms. Subjects treated with PZQ 40 mg/kg were at higher risks compared to PZQ 2*30 mg/kg (RR 14.10, 95%CI 1.92–103.68, p = 0.009) and oxamniquine 40 mg/kg (RR 0.03, 95%CI 0.01–0.19, p = 0.001). The risk was also higher with Biltricide than Distocide (RR 2.28, 95%CI 1.46–3.56, p = 0.001), while there was no difference between PZQ 2*20 mg/kg (6%) and PZQ 2*15mg/kg (1%) and PZQ 2*25 mg/kg (5%), or between PZQ 40 mg/kg tablet and syrup formulation, or between 40 mg/kg and other PZQ doses, artesunate combinations, metrifonate 10 mg/kg, mefloquine, and other oxamniquine doses.

The incidence of dizziness was 11.9% (95%CI 7.9–16.2) in 5,522 PZQ 40 mg/kg recipients enrolled in 26 treatment arms. Subjects treated with PZQ 40 mg/kg were at lower risks than oxamniquine at any dose: 20 mg/kg (RR 0.31, 95%CI 0.21–0.48, p = 0.001), 25 mg/kg (RR 0.56, 95%CI 0.37–0.84, p = 0.005), 30 mg/kg (RR 0.21, 95%CI 0.14–0.32, p = 0.001), 40 mg/kg (RR 0.19, 95%CI 0.13–0.27, p = 0.001), while they were at higher risks compared to metrifonate 10 mg/kg (RR 1.60, 95%CI 1.06–2.43, p = 0.001); there was no difference between the different dose of PZQ treatment and syrup, L-PZQ, oltripaz 2*15 mg/kg, metrifonate 30 mg/kg, or niridazole 150 mg/kg.

Nausea was reported in 10.6% (95%CI 6.8–14.9) in 5,824 PZQ 40 mg/kg subjects in 22 treatment arms. Subjects treated with PZQ 40 mg/kg were at higher risks for nausea compared to 2*30 mg/kg (RR 2.47, 95%CI 1.18–5.16, p = 0.001) and L-PZQ (RR 4.50, 95%CI 1.56–12.96, p = 0.001), while there was no difference between the different dose of PZQ (20, 25, 30, 2*15, 40, 2*25, 60, 2*35, 80 mg/kg), brands and formulations, artesunate and combinations, oltripaz 2*15 mg/kg, metrifonate 10, 30 mg/kg, oxamniquine (15, 20, 25, 30, 40 mg/kg), niridazole 150 mg/kg.

The incidence of itching/rash was 9.8% (95%CI 3.8–18.2) in 3,340 PZQ 40 mg/kg recipients in 16 treatment arms. Splitting the dose (PZQ 2*20 mg/kg) decreased the risk of itching/rash (RR 0.03, 95%CI 1.01–0.52, p = 0.016) in one study; no difference was detected between PZQ brands, tablets vs. syrup, and between PZQ 40 mg/kg and 2*30, 2*25 mg/kg, metrifonate 10 and 30 mg/kg, oxamniquine at various doses (20, 25, 30, 50 mg/kg), artesunate combinations and niridazole 150 mg/kg.

Fatigue was reported in 9.6% (95%CI 4.0–16.3) of the 2,595 PZQ 40 mg/kg recipients in 10 arms. Subjects treated with PZQ 40 mg/kg were at lower risks compared to oxamniquine 25 mg/kg (RR 0.17, 95%CI 0.05–0.58, p = 0.005) while there was no difference between PZQ 40 mg/kg compared to other doses of PZQ (2*15, 2*20, 2*25, 2*30 mg/kg), between PZQ brands, formulations, L-PZQ, oxamniquine 15 mg/kg, and metrifonate 10 mg/kg.

The incidence of vomiting was 7.9% (95%CI 5.2–10.9) in 5,722 PZQ 40 mg/kg recipients enrolled in 27 treatment arms. Subjects treated with PZQ 40 mg/kg were at lower risks compared to 60 mg/kg (RR 0.44, 95%CI 0.26–0.72, p = 0.001) but at higher risk than PZQ 2*30 mg/kg (RR 2.51, 95%CI 1.26–4.97, p = 0.008); the risk was higher with Biltricide than Distocide (RR 3.53, 95%CI 1.88–6.63, p = 0.001). There was no difference between tablets and syrup, and between 40 mg/kg and other doses (20, 30, 2*20 mg/kg), L-PZQ, AS, ASSP, ASMQ, oxamniquine (15, 20, 25, 30 mg/kg) or metrifonate 3*10 mg/kg.

## Discussion

This is, to our knowledge, the largest collection of PZQ treatment trials analyzed so far, with over 14,000 subjects receiving the drug at different doses. This population is much larger, but intrinsically less homogenous, than that of the two available Cochrane systematic reviews [6], [7]. This study complements the Cochrane systematic reviews by broadening the number of studies analyzed for efficacy as well as tolerability, and by allowing a side-by-side analysis of all *Schistosoma* species, including co-infections. The overall conclusions of these reviews are generally concordant, despite some minor differences, which are mostly related to the different criteria for including/excluding studies in either analyses. Provided basic methodological standards are guaranteed, relaxing eligibility criteria for meta-analysis to allow for both comparative and non-comparative trials can broaden the database and complement more classical meta-analysis of randomised controlled trials.

This and the Cochrane meta-analyses point to the lack of methodological standardization of the studies analyzed: the number of subjects per study, age groups, species and PZQ doses varied greatly across the studies; different methodologies were used to detect eggs in excreta (number of samples taken; number of tests/sample) and to quantify efficacy (CR, ERR with arithmetic or geometric means). While studies extend over three decades and a range of countries, trends over time cannot be reliably derived. In 38% of the studies, patient attrition was greater than 10%, and this more in community-based (45%) than school-based studies (31%). Tolerability was unevenly assessed and reported. Statistical models have helped in deriving trends but cannot compensate for the lack of direct comparisons for dose and age effects.

On the other hand, the large number of subjects allows generalizable conclusions, despite the inherent limitations of aggregated-data meta-analyses.

The main findings of this meta-analysis are that: (1) *Schistosoma* species appear to respond differently to PZQ, with *S. japonicum* having the highest and mixed *S. mansoni/haematobium* infections the lowest response rates, both in terms of CR and ERR; (2) a dose-response trend was apparent for CR in *S. mansoni* and mixed *S. mansoni/haematobium* infections, but not *S. haematobium* or *S. japonicum*. No significant trend was apparent for ERR, the currently preferred outcome measure [4] for any of the *Schistosoma* species; (3) age did not appear to influence treatment outcomes. However, this should be interpreted with caution as the age groups enrolled were generally broad and details by age are generally not provided in the papers; furthermore, preschool-aged children are minimally represented in this population, received only 40 mg/kg, and only for *S. mansoni*; (4) a single praziquantel dose of 40 mg/kg appears a reasonable compromise for all species and ages, although in a proportion of cases efficacy may be lower than expected.

The most studied groups were school-aged children (64% of all subjects), *S. mansoni* infections (58%) and the PZQ dose of 40 mg/kg (56%); 68% of subjects were in the WHO AFRO region (where the prevalence of the infection is highest). Preschool-aged children accounted for only ∼3% of the total population (meaning that information on younger children is limited, and that conclusions on age-related efficacy and safety may change when more data accumulate in this age group). It should also be noted that community-based studies (which generally enroll subjects of all ages) tend to have more drop-outs than school-based studies.

Overall, the CR achieved with the WHO-recommended dose of 40 mg/kg was highest for *S. japonicum* (94.7%, 95%CI 92.2–98.0), followed by *S. haematobium* (77.1%, 95% CI 68.4–85.1) for *S. haematobium*, and *S. mansoni* (76.7%, 95% CI 71.9–81.2), and mixed *S. haematobium* and *S. mansoni* infections (63.5%, 95%CI 48.2–77.0). Recent WHO Standard Operating Procedures recommend that control programs should further investigate drug performance in populations where the ERR is found to be lower than 90% [4]. The average ERR obtained in school-aged children with the dose of 40 mg/kg was 95% for *S. japonicum*, 94% for *S. haematobium*, and 89% for *S. mansoni*. Since these values are derived from a collection of studies, the fact that the lower bound of the 95%CI (obtained by bootstrapping) was 90% for *S. japonicum*, 89% for *S. haematobium*, and 81% for *S. mansoni* means that a proportion of these sites might warrant further assessment. However, it is difficult to compare results obtained with a variety of diagnostic approaches and using different calculations (geometric or arithmetic means) to a ‘reference for drug efficacy’ that is based on a single examination of a single specimen and is expressed as geometric mean.

Praziquantel efficacy may be influenced by a variety of factors, which could not be explored in detail using aggregated data and meta-analysis methods. Pre-treatment intensity of infection is one, which could not be fully accounted for by having it as covariate in the model, primarily because of the diversity of the diagnostic and calculation approaches used, and because it is reported as group mean (individual subject data meta-analyses are better suited to address this issue).

The WHO-recommended dose of 40 mg/kg compared favorably to all other PZQ regimens and other treatments tested. The dose-response curve appears to be flat for *S. haematobium* and to plateau at 40 mg/kg for *S. mansoni*. This must be due to different species susceptibility, because this happens in spite of exposure to praziquantel increasing overproportionally with the dose (the first-pass-metabolism in the liver being dose-dependent with regard to capacity) [84]. Similar to the Cochrane review [7], oxamniquine at 40 and 50 mg/kg appears to be an effective, but less well tolerated, alternative limited however to *S. mansoni*, and no longer available in the WHO AFRO and EMRO regions.

We provide an extensive report of safety findings. Tolerability was variably assessed in 12,435 subjects enrolled in 40 studies. Safety information was provided for 45% (25/55) of the studies included in the efficacy meta-analysis; we identified an additional 15 studies with safety information which were not included in the efficacy analysis. Reporting on safety was highly variable, and we cannot confidently conclude whether the absence of a given AE in a certain study means that it did not occur or it was not investigated. Lastly, the frequencies reported must be taken separately for each individual AE (they should not be accumulative), as some subjects might have experienced more than one AE. From comparative studies, risk seems not to change with the PZQ dose overall, although there are indications that higher doses may induce more events in some cases (e.g. 60 mg/kg had more of any AE and vomiting than 40 mg/kg; more abdominal pain with 40 mg/kg than 20 mg/kg), and that a split dose (20 mg/kg twice) may be better tolerated (in particular fewer cases of itching/rash).

A direct comparison of two brands of praziquantel (Biltricide and Distocide) found the former to cause more AEs (abdominal pain, fever, diarrhea, headache, vomiting). The reason could be related to higher blood levels: when the pharmacokinetics of these two brands given at 40 mg/kg to healthy volunteers was compared, Biltricide peak concentration (Cmax) was 1.9 times higher (mean 1.281 vs. 0.685 µg/ml) and the area under the concentration curve (AUC) was 1.7 (mean 3550 vs. 2133 ng/h/ml) times higher than Distocide [47].

One limitation of the tolerability analysis relates to the diversity of the definitions of ‘events’ and methods to express incidence rates across studies. Therefore we opted for a permissive definition allowing for any sign or symptom occurring after treatment, acknowledging that this may indeed overestimate the real contribution of the treatment to the occurrence of events. Moreover information on signs or symptoms and their severity before treatment was only collected in a few studies so that it was not possible to detect which events were treatment-emergent.

The adoption of more standardized methodologies in clinical studies would facilitate meta-analyses and strengthen the quality of evidence, as already pointed out for urinary schistosomiasis [85]; some of these questions can be answered more adequately only through an individual-subject data meta-analysis.

**Box 1**

- This is the largest collection of trials on praziquantel for treating urinary and intestinal schistosomiasis which has been meta-analysed for efficacy and safety
- Provided basic methodological standards are guaranteed, relaxing eligibility criteria for meta-analysis to allow for both comparative and non-comparative trials can broaden the database and complement more classical meta-analysis of randomised controlled trials
- Results support World Health Organization recommendations and are consistent with Cochrane systematic reviews

**Box 2**

- Steinmann P, Keiser J, Bos R, Tanner M, Utzinger J (2006) Schistosomiasis and water resources development: systematic review, meta-analysis, and estimates of people at risk. Lancet Infect Dis 6: 411–25.
- World Health Organization (2013) Assessing The Efficacy Of Anthelminthic Drugs Against Schistosomiasis And Soil-Transmitted Helminthiases. Available: http://apps.who.int/iris/bitstream/10665/79019/1/9789241564557_eng.pdf. Accessed 2/1/2014.
- DerSimonian R, Laird N (1986) Meta-analysis in clinical trials. Control Clin Trials 7: 177–88.
- Pocock SJ, Travison TG, Wruck LM (2008) How to interpret figures in reports of clinical trials. BMJ 336: 1166–9.
- Kramer CV, Zhang F, Sinclair D, Olliaro PL (2014) Drugs for treating urinary schistosomiasis. Cochrane Database Syst Rev 8: CD000053.

## Supporting Information

Checklist S1 **Prisma checklist.**
(PDF)

Figure S1 **Flow chart of the number of studies and patients screened and eligible for the safety analysis.**
(BMP)

Table S1 **Number of patients enrolled by country and species (all treatment arms).**
(PDF)

Table S2 **Study design of the included publications: risks of bias and attrition.**
(PDF)

Table S3 **Classing of studies by age groups based on reported age ranges.**
(PDF)

Table S4 **Diagnostic approaches used in the studies (number of study/sites).**
(PDF)
