# Supplementary figures and images for: Clinical Efficacy and Tolerability of Praziquantel for Intestinal and Urinary Schistosomiasis—A Meta-analysis of Comparative and Non-comparative Clinical Trials
Source: PLoS Negl Trop Dis. 2014 Nov 20;8(11):e3286. doi: 10.1371/journal.pntd.0003286 (PMC4238982; doi:10.1371/journal.pntd.0003286)

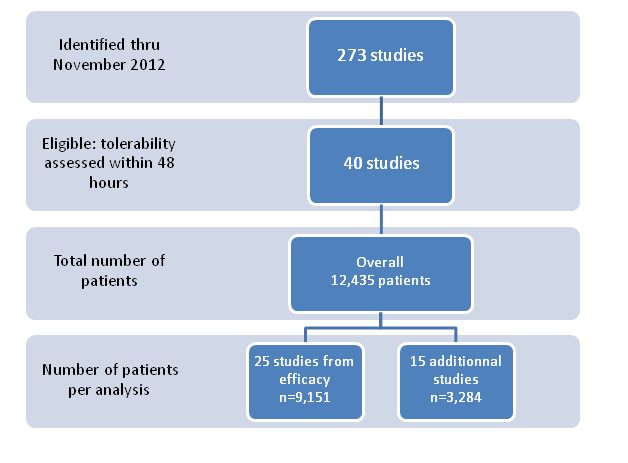

Supplement: Figure S1 — Flow chart of the number of studies and patients screened and eligible for the safety analysis. (BMP) [file pntd.0003286.s002.bmp]
