# Supplementary material for: Clinical Efficacy and Tolerability of Praziquantel for Intestinal and Urinary Schistosomiasis—A Meta-analysis of Comparative and Non-comparative Clinical Trials
Source: PLoS Negl Trop Dis. 2014 Nov 20;8(11):e3286. doi: 10.1371/journal.pntd.0003286 (PMC4238982; doi:10.1371/journal.pntd.0003286)
Supplement: Table S1 — Number of patients enrolled by country and species (all treatment arms). (PDF) [file pntd.0003286.s003.pdf]

Supporting information Table S1. Number of patients enrolled by country and species (all treatment arms)

Species / Country	*S. haematobium*	*S. haematobium + intercalatum*	*S. japonicum*	*S. mansoni*	*S. mansoni + haematobium*	Total by country	% of total countries
Egypt				2606		2606	13.4%
Kenya	719			1597		2316	11.9%
Sudan				1375	374	1749	9.0%
Uganda				1283		1283	6.6%
Ethiopia				1230		1230	6.3%
Burundi				1138		1138	5.8%
Zimbabwe	675				373	1048	5.4%
Ivory coast	523			453		976	5.0%
Senegal	288			558		846	4.3%
Mali	800					800	4.1%
Philippines, China			793			793	4.1%
Cameroon	674					674	3.5%
Gabon	465	158				623	3.2%
Gambia	619					619	3.2%
Tanzania	228			373		601	3.1%
Philippines			409			409	2.1%
Nigeria	402					402	2.1%
Brazil				316		316	1.6%
Niger	208					208	1.1%
China			205			205	1.1%
Mauritania				186		186	1.0%
Saudi Arabia				170		170	0.9%
Zambia	151					151	0.8%
Botswana				81		81	0.4%
Malawi				69		69	0.4%
Total by species	5752	158	1407	11435	747	19499	100%
% of all species	29.5%	0.8%	7.2%	58.6%	3.8%	100%	
