# Supplementary material for: Clinical Efficacy and Tolerability of Praziquantel for Intestinal and Urinary Schistosomiasis—A Meta-analysis of Comparative and Non-comparative Clinical Trials
Source: PLoS Negl Trop Dis. 2014 Nov 20;8(11):e3286. doi: 10.1371/journal.pntd.0003286 (PMC4238982; doi:10.1371/journal.pntd.0003286)
Supplement: Table S2 — Study design of the included publications: risks of bias and attrition. (PDF) [file pntd.0003286.s004.pdf]

Supporting information Table S2. Study design of the included publications: risks of bias and attrition

Species	Publication (name, year)	Country	Endpoint (month)	Age group	Attrition				Study design		
					Enrolled (n)	Assesed (n)	Assessed (%)	Risk of bias	Control	Blinding	Details
sh	Bornmann 2001	Gabon	2	school	300	296	99%	low	cs		ma, community based, computer-generated randomization, other risks of randomisation bias unclear
	Burchard 1984	Gabon	2	school	165	138	84%	high	cs		school based, risks of randomisation bias unclear
	Davis 1981	Zambia	1	school	151	151	100%	low	cs	2	ma, school based, Allocation concealment: double blind, low risks of randomisation bias
	de Clercq 2002	Senegal	2	school	288	267	93%	low	cs		school based, exhaustive, selective treatment study, unclear risks of randomisation bias
	Inyang-Etoh 2008	Nigeria	2	school	312	262	84%	high	cs		school based, exhaustive, placebo controlled, randomisation unclear
	Keiser 2010	Ivory coast	1	school	83	83	100%	low	cs		school based, exploratory open-label trial, computer-generated randomization code, unclear risks of other randomisation bias
	King 2002	Kenya	2	all ages	291	200	69%	high	cs		ma, school based, computer-generated sequence, unclear risks of other randomisation bias
	Latham 1990	Kenya	2	school	48	48	100%	low	cs		school-based, male only, unclear risks of randomisation bias
	McMahon 1983	Tanzania	2	all ages	138	125	91%	low	cs		ma, school based, exhaustive, stratified randomisation, unclear risks other randomisation bias
	McMahon 1979	Tanzania	1	school	90	77	86%	high	cs		ma, unclear risks of randomisation bias
	Midzi 2008	Zimbabwe	2	school	675	624	92%	low	sts	na	school based, exhaustive, selective treatment study
	N'goran 2003	Ivory coast	1	school	440	354	80%	high	sts	na	School based, exhaustive, selective treatment study
	Oyideran 1981	Nigeria	1	school	90	82	91%	low	cs		ma, placebo controlled, unclear risks of other randomisation bias
	Rey 1983	Niger	1	young adult	208	188	90%	low	cs		ma, school based, unclear risks of randomisation bias
	Sissoko 2009	Mali	1	school	800	781	98%	low	cs	1	School based, block randomization, single blind, high quality
	Tchuente 2004	Cameroon	1	school	674	515	76%	high	sts	na	school based, exhaustive, selective treatment study
	Wilkins 1987	Gambia	1	school	619	619		na	sts	na	ma, computer-generated, unclear risks of randomisation bias and attrition bias incomplete
sh + si	Kern 1984	Gabon	2	school	158	158		na	cs		exhaustive, school based, unclear risks of randomisation bias
sj	Belizario 2007	Philippines	1	school	206	203	99%	low	cs	2	school based, randomized, double blind
	Hou 2008	China	2	all ages	205	196	96%	low	cs	2	hospital based, randomized, double-blind, placebo-controlled, other bias unclear
	Olliaro 2011	Philippines	1	school	203	200	99%	low	cs	2	ma, double-blind trial, computerised block size of 4, low risk of bias
sj/sm/ sh	Olds 1999	Kenya, Phillipines,	2	school	1540	1540	100%	low	cs	2	school based, double blind placebo controlled, multisite, randomised block design of size 80, low risk of bias

		China									
sm	Abu elyazed 1998	Egypt	2	all ages	975	939	96%	low	sts	na	community based, exhaustive, 2 selective treatment studies
	Barakat 2005	Egypt	1	all ages	104	83	80%	high	cs		community based, exhaustive, randomized in two groups, the first for Myrrh and the second for praziquantel, unclear risks of randomisation bias
	Berhe 1999	Ethiopia	2	school	611	541	89%	high	sts	na	school based, exhaustive, selective treatment study
	Botros 2005	Egypt	1,2	all ages	379	271	72%	high	cs		community based, stratified randomisation, unclear risks of randomisation bias
	daSilva 1986	Brazil	1	all ages	120	94	78%	high	cs	2	ma, based not reported, double blind, unclear risks of other randomisation bias
	Declerq 2000b	Senegal	2	all ages	180	156	87%	high	cs		hospital based, unclear risks of randomisation bias
	Declerq tmih 2000	Senegal	2	all ages	110	110	100%	low	cs		ma, community based, unclear risks of randomisation bias
	Degu 2002	Ethiopia	2	school	154	148	96%	low	sts	na	school based, exhaustive, selective treatment study
	Friis 1988	Botswana	2	school	81	81	100%	low	sts	na	school based, not exhaustive, selective treatment study, stratified randomisation
	Ghandour 1995	Saudi Arabia	1	all ages	170	170	100%	low	sts	na	community based, exhaustive, selective treatment study
	Gryseels 1987	Burundi	2	all ages	1138	1049	92%	low	sts	na	community based, exhaustive, selective treatment study
	Guisse 1987	Senegal	1	school	130	130	100%	low	cs		ma, community based, unclear risks of randomisation bias and attrition bias incomplete
	Homeida 1989	Sudan	2	all ages	885	806	91%	low	cs	1	ma, community based, single blind, unclear risks of randomisation bias
	Ismail 1994	Egypt	2	all ages	463	436	94%	low	sts	na	community based, exhaustive, selective treatment study
	Kabatereine 2003	Uganda	2	all ages	617	482	78%	high	sts	na	community based, not exhaustive, selective treatment study
	Kardaman 1983	Sudan	1	all ages	388	347	89%	high	sts	na	ma, community based, exhaustive, unclear risks of randomisation bias
	Massoud 1984	Egypt	1	school	179	179	100%	low	cs		school based, exhaustive, unclear risks of randomisation bias
	McMahon 1981	Tanzania	1	all ages	102	91	89%	high	cs		ma, community based, exhaustive, unclear risks of randomisation bias
	Metwally 1995	Egypt	1	school	506	366	72%	high	cs		school based, exhaustive, stratified randomisation, unclear risks of randomisation bias
	Mohamed 2009	Sudan	1	school	102	92	90%	low	cs		school based, open label, unclear risks of randomisation bias
	Navaratnam 2012	Uganda	1	preschool	297	203	68%	high	cs		community based, exhaustive, every second child assigned to the same treatment arm, other risks of randomisation bias unclear
	Obonyo 2010	Kenya	1	school	212	204	96%	low	cs	1	school based, open-label randomised trial, computer-generated block, single blind, unclear other risks of bias
	Olliaro 2011	Brazil	1	all ages	196	190	97%	low	cs	2	ma, double-blind trial, computerised block size of 4, sealed and numbered envelopes
	Olliaro 2011	Mauritania	1	all ages	186	185	99%	low	cs	2	ma, double-blind trial, computerised block size of 4,

											sealed and numbered envelopes
	Olliaro 2011	Tanzania	1	school	271	244	90%	low	cs	2	ma, double-blind trial, computerised block size of 4, sealed and numbered envelopes
	Raso 2004	Ivory coast	2	all ages	200	161	81%	high	sts	na	community based, exhaustive, selective treatment study
	Simonsen 1990	Ethiopia	1	school	265	206	78%	high	sts	na	school based, exhaustive, selective treatment study
	Sousa-Figueiredo 2012	Uganda	1	preschool	369	305	83%	high	sts	na	community based, exhaustive, selective treatment study
	Stelma 1997	Senegal	2	all ages	138	138	100%	low	cs		community based, stratified randomisation, unclear risk of bias
	Taddese 1988	Ethiopia	1	adult	200	191	96%	low	cs		ma, farm based, randomly assigned, unclear risk of bias
	Teesdale 1984	Malawi	1	all ages	69	69	100%	low	cs		ma, community based, exhaustive, stratified randomisation, unclear risk of other bias
	Thiongo'o 2002	Kenya	2	school	1018	1018		na	cs		school based, exhaustive, unclear risk of randomisation bias and attrition bias incomplete
	Utzinger 2000 tmih	Ivory coast	1	school	253	194	77%	high	sts	na	school based, selective treatment study, unclear risk of randomisation bias
sm+sh	El Tayeb 1988	Sudan	1	school	111	111	100%	low	cs		school based, exhaustive, randomisation on the toss of a coin, unclear other risks of randomisation bias
	Kardaman 1983	Sudan	1	all ages	43	37	86%	high	sts	na	community based, exhaustive, selective treatment study
	Kardaman 1985	Sudan	2	school	373	373	100%	low	sts	na	ma, school based, exhaustive, unclear risks of randomisation bias
	Taylor 1988	Zimbabwe	1	school	220	211	96%	low	cs	1	ma, school based, exhaustive, single blind, unclear risk of other bias

Legend: Blinding, 1: single, 2: double; endpoint, 1:within one month, 2: within two months; sm, *S. mansoni*; sh, *S. haematobium*; sj, *S. japonicum*; cs, comparative study; sts, selective treatment study; na, not applicable; ma, included in *S. mansoni* or *S. haematobium* meta-analysis; Exhaustive: all people screened whether community or school based study; Selective treatment study: non-comparative study using praziquantel; The attrition risk bias is measured as the number of patients not assessed at endpoint time out of the number of patients enrolled and considered high when greater than 10%.
