# Supplementary material for: Clinical Efficacy and Tolerability of Praziquantel for Intestinal and Urinary Schistosomiasis—A Meta-analysis of Comparative and Non-comparative Clinical Trials
Source: PLoS Negl Trop Dis. 2014 Nov 20;8(11):e3286. doi: 10.1371/journal.pntd.0003286 (PMC4238982; doi:10.1371/journal.pntd.0003286)
Supplement: Table S3 — Classing of studies by age groups based on reported age ranges. (PDF) [file pntd.0003286.s005.pdf]

Supporting information Table S3. Classing of studies by age groups based on reported age ranges.

Species	Publication	Country	Age group	Age range	Total enrolled (n)
S. haematobium	Bormann 2001	Gabon	school	5-13	300
	Burchard 1984	Gabon	school	5-14	165
	Davis 1981	Zambia	school	7-17	151
	de Clercq 2002	Senegal	school	7-14	288
	Inyang-Etoh 2008	Nigeria	school	4-20	312
	Keiser 2010	Ivory coast	school	8-16	83
	King 2002	Kenya	all	4-23	291
	Latham 1990	Kenya	school	7-15	48
	McMahon 1979	Tanzania	school	7-15	138
	McMahon 1983	Tanzania	all	1-65	90
	Midzi 2008 a	Zimbabwe	school	2-19	675
	N'goran 2003	Ivory coast	school	5-15	440
	Oyideran 1981	Nigeria	school	7-13	90
	Rey 1983	Niger	adult	15-20	208
	Sissoko 2009	Mali	school	6-15	800
	Tchuente 2004	Cameroon	school	na	674
	Wilkins 1987	Gambia	school	5-17	619
S. haematobium + intercalatum	Kern 1984	Gabon	school	10-17	158
S. japonicum	Belizario 2007	Philippines	school	10-19	206
	Hou 2008	China	all	10-60	205
	Olliaro 2011	Philippines	school	7-12	203
S. japonicum / S. haematobium	Olds 1999	Philippines, China, Kenya	school	5-19	1540
S. mansoni	Abu Elyazed 1998	Egypt	all	5-50	975
	Barakat 2005	Egypt	all	5-39	104
	Berhe 1999	Ethiopia	school	5-17	611
	Botros 2005	Egypt	school and all	12-17 and 7-73	379
	daSilva 1986	Brazil	all	14-65	120
	Declerq 2000	Senegal	all	6-61	180
	Declerq tmih 2000	Senegal	all	1-50	110
	Degu 2002	Ethiopia	school	10-14	154
	Friis 1988	Botswana	school	na	81
	Ghandour 1995	Saudi Arabia	all	1-50	170
	Gryseels 1987	Burundi	adult	<20 and >20	1138
	Guisse 1987	Senegal	school	5-15	130
	Homeida 1989	Sudan	all	1-65	885
	Ismail 1994	Egypt	all	6-18	463
	Kabatereine 2003	Uganda	all	5-65	617
	Kardaman 1983	Sudan	all	5-65	388
	Massoud 1984	Egypt	school	primary	179
	McMahon 1981	Tanzania	all	1-65	102
	Metwally 1995	Egypt	school	8-16	506
	Mohamed 2009	Sudan	school	8-17	102
	Navaratnam 2012	Uganda	Pre-school	1-5	297
	Obonyo 2010	Kenya	school	7-12	212
	Olliaro 2011	Brazil	all	10-19	196
	Olliaro 2011	Mauritania	all	10-19	186
	Olliaro 2011	Tanzania	school	10-19	271
	Raso 2004	Ivory coast	all	1-65	200
	Simonsen 1990	Ethiopia	school	5-14	265
	Sousa-Figueiredo 2012	Uganda	Pre-school	1-7	369
	Stelma 1997	Senegal	all	5-75	138
	Taddese 1988	Ethiopia	adult	17-52	200
	Teesdale 1984	Malawi	all	9-15	69

	Thiongo'o 2002	Kenya	school	5-17	1018
	Utzinger 2000	Ivory coast	school	6-14	253
S. mansoni + haematobium	El Tayeb 1988	Sudan	school	7-12	111
	Kardaman 1983	Sudan	all	5-65	43
	Kardaman 1985	Sudan	school	7-11	373
	Taylor 1988	Zimbabwe	school	10-15	220
