# Supplementary material for: Clinical Efficacy and Tolerability of Praziquantel for Intestinal and Urinary Schistosomiasis—A Meta-analysis of Comparative and Non-comparative Clinical Trials
Source: PLoS Negl Trop Dis. 2014 Nov 20;8(11):e3286. doi: 10.1371/journal.pntd.0003286 (PMC4238982; doi:10.1371/journal.pntd.0003286)
Supplement: Table S4 — Diagnostic approaches used in the studies (number of study/sites). (PDF) [file pntd.0003286.s006.pdf]

Supporting Information 4. Diagnostic approaches used in the studies (number of study/sites)

Diagnostic approach	*S. haematobium*	*S. haematobium + intercalatum*	*S. japonicum*	*S. mansoni*	*S. mansoni + haematobium*	Total
1 slide / 1 sample	3			7	1	11
1 slide / 1 sample (duplicate)	3			7		10
1 slide / 2 samples	5	1		3	1	10
1 slide / 2 samples (duplicate)			1	3		4
1 slide / 3 samples	3			1		4
1 slide / 3 samples (duplicate)				1		1
1 slide / 4 samples				1		1
2 slides / 1 sample	2		1	3	1	7
2 slides / 1 sample (triplicate)			1			1
2 slides / 2 samples	2		1	2		5
3 slides / 1 sample				4	1	5
3 slides / 3 samples	1			1		2
3 slides / 3 samples (triplicate)				2		2
4 slides / 1 sample				1		1
not specified				1		1
**total by species**	**19**	**1**	**4**	**37**	**4**	**65**
